# Functional Dissection of SseF, a Membrane-Integral Effector Protein of Intracellular *Salmonella enterica*

**DOI:** 10.1371/journal.pone.0035004

**Published:** 2012-04-18

**Authors:** Petra Müller, Deepak Chikkaballi, Michael Hensel

**Affiliations:** 1 Mikrobiologisches Institut, Universitätsklinikum Erlangen, Erlangen, Germany; 2 Abteilung Mikrobiologie, Universität Osnabrück, Osnabrück, Germany; University of Birmingham, United Kingdom

## Abstract

During intracellular life, the bacterial pathogen *Salmonella enterica* translocates a complex cocktail of effector proteins by means of the SPI2-encoded type III secretions system. The effectors jointly modify the endosomal system and vesicular transport in host cells. SseF and SseG are two effectors encoded by genes within *Salmonella* Pathogenicity Island 2 and both effector associate with endosomal membranes and microtubules and are involved in the formation of *Salmonella*-induced filaments. Our previous deletional analyses identified protein domains of SseF required for the effector function. Here we present a detailed mutational analysis that identifies a short hydrophobic motif as functionally essential. We demonstrate that SseF and SseG are still functional if translocated as a single fusion protein, but also mediate effector function if translocated in cells co-infected with *sseF* and *sseG* strains. SseF has characteristics of an integral membrane protein after translocation into host cells.

## Introduction

*Salmonella enterica* is a facultative intracellular pathogen with the ability to create a unique compartment in host cells, termed *Salmonella*-containing vacuole (SCV). The SCV has certain characteristics of late endosomal compartments, but does not undergo final maturation to phagolysosomes. Within the SCV, *Salmonella* appears protected against antimicrobial effectors of the host and can efficiently proliferate (reviewed in [1]). Various virulence determinants are required for the adaptation to this intracellular habitat, but of central importance is the type III secretion system (T3SS) encoded by *Salmonella* Pathogenicity Island 2 (SPI2) [2]. The SPI2-T3SS is active in *Salmonella* residing within the SCV and translocates a cocktail of 20 and possibly more effector proteins across the SCV membrane [3].

The intracellular lifestyle of *Salmonella* is accompanied by a number of unique phenotypical alterations to the host cell. The SCV behaves like a novel organelle, and SPI2-T3SS function is required to maintain the positioning of the SCV in a subcellular localization that is permissive for proliferation [4], [5], [6]. The redirection of host cell vesicular trafficking is dependent on the SPI2 function and the most dramatic phenotype is the massive remodeling of the host cell endosomal system that results in the aggregation of endosomal vesicles to large tubular structures referred to as *Salmonella*-induced filaments, or SIF [7]. SIF are characterized by the presence of lysosomal glycoproteins and recent studies showed that SIF are highly dynamic structures that extend and collapse [8], [9]. The extension of SIF required the integrity of the microtubule cytoskeleton [10], [11].

The molecular targets for most of the SPI2-T3SS effector proteins are not known, and mutational analyses indicated that only a subset of these proteins is required to maintain the SCV and to enable intracellular proliferation of *Salmonella*. These effectors, SifA, SseF, SseG, PipB2 and SopD2 share a common subcellular localization after SPI2-T3SS-dependent translocation, and can be found in close association with the membrane of SCV and SIF.

Mutant strains deficient in *sifA* have the most severe virulence defect *in vivo* and on the cellular level, the mutant strains fail to induce SIF and to modify vesicular trafficking [12]. *sifA* strains are unable to maintain the SCV and escape into the host cell cytoplasm [13]. SifA is attached to endosomal membranes by a C-terminal prenylation motif [14]. PipB2 acts as a linker for microtubule motor complex kinesin [15] and a reduced centripedal growth of SIF was observed for *pipB2* strains [16]. The molecular function of SopD2 has not been characterized in larger detail.

SseF and SseG are effector proteins encoded by genes within SPI2 and may belong to the ancestral set of effectors that was complemented by further effectors present on further genetic loci outside of SPI2. SseF and SseG are both associated with the SCV membrane as well as with the membranes of SIF [17]. Both SseF and SseG are characterized by large hydrophobic domains that may be responsible for the interaction of these effectors with host cell membranes. Defects in either SseF or SseG result in a moderate reduction of systemic pathogenesis and attenuation of intracellular proliferation. In cells infected with *sseF* or *sseG* mutant strains, the overall induction of SIF is reduced and SIF show an aberrant morphology, termed ‘pseudo-SIF’ [17]. Pseudo-SIF are characterized by a ‘beads on a string’-like appearance in fixed host cells that may indicate a more fragile structure of the endosomal aggregates compared to SIF induced by WT *Salmonella*. Both effectors also contribute to the positioning of the SCV to a juxtanuclear, Golgi-associated subcellular localization [18], [19].

We have previously characterized SseF and initiated a functional dissection of this effector protein [19]. Deletions of various domains of SseF indicated that the first hydrophobic regions in the N-terminal part of the protein is required for the translocation by the T3SS while the second hydrophobic part in the C-terminal moiety of SseF is required for the effects on the host cell [19].

In this study, we investigated the topology of SseF after translocation into host cells, characterized functional domains and the interaction with other SPI2 effector proteins. We observed that translocated SseF has properties of an integral membrane protein in endosomal membranes.

## Results

### Functionally essential regions of SseF identified by mutational analysis

**Figure 1:**
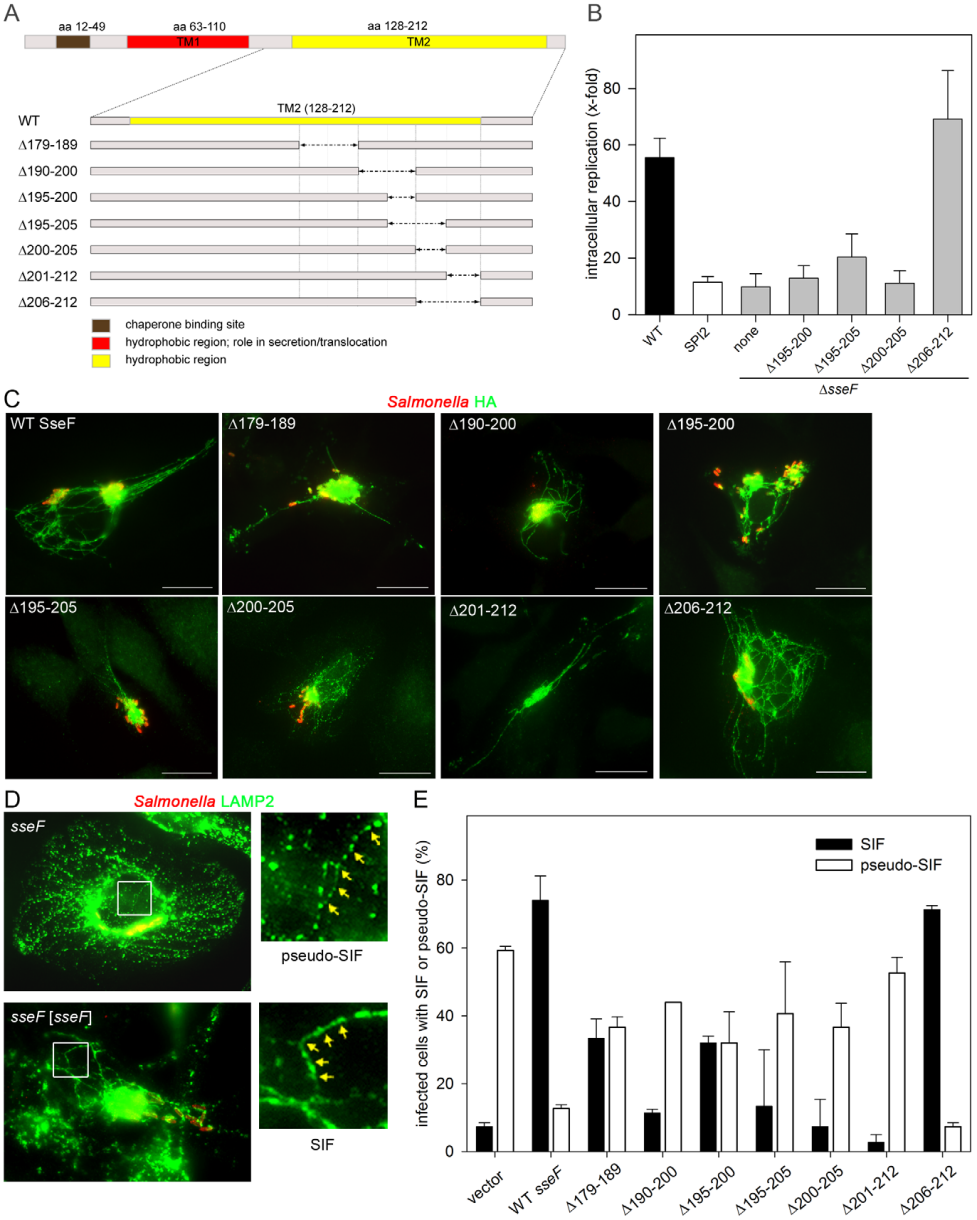
Functional dissection of the C-terminal hydrophobic domain of SseF. A) Location of in-frame deletions of various extents in the C-terminal moiety of SseF. Functions of domains in SseF as revealed by previous studies [19], [34] are indicated. B) Analyses of intracellular replication in HeLa cells of *Salmonella* WT, a SPI2 null mutant strain, and the *sseF* strain without or with plasmids for the expression of various mutant alleles of *sseF*. The amount of intracellular colony-forming units (CFU) was determined 2 h and 16 h after infection and intracellular replication is the ratio of CFU at 16 h/2 h. Means and standard deviation of three assays are shown. C) Translocation of deletion variants of SseF. HeLa cells were infected with the *sseF* strain harboring plasmids for the expression of *sseF*::HA or various mutant alleles of *sseF* as indicated. Cells were fixed 16 h p.i. and immuno-stained for SseF-HA (detected with α rat Alexa488, green) and LPS (detected with α rabbit Alexa568, red). Scale bars: 20 µm. D, E) SIF phenotypes in cells after translation of various SseF variants. HeLa cells were infected with the *sseF* strain without plasmid or with plasmids for the expression of WT *sseF* or various mutant alleles as indicated. Cells were fixed 16 h after infection and immuno-stained for *Salmonella* LPS (red) and LAMP2 (green). The formation of SIF or pseudo-SIF in infected host cells was scored. D) Representative cells showing SIF or pseudo-SIF formation are shown. The white frame was enlarged and arrows indicate the typical appearance of SIF and pseudo-SIF. E) Quantification of SIF and pseudo-SIF formation by the various deletion strains. At least 50 infected cells were identified and the percentage of cells showing SIF (filled bars) or pseudo-SIF (open bars) was calculated. The means and standard deviations of three independent experiments are shown.

In our previous study [19], the second hydrophobic domain of SseF turned out to be important for many effector functions of SseF. More specifically, this region could be mapped to 33 amino acids (179–212) present in the C-terminal part of the second hydrophobic region [19]. In order to further characterize the domain responsible for the effector functions, a second round of deletions within this 33 amino acid motif was performed. The various deletion mutants are represented in Fig. 1A. In order to detect all variants of SseF, the HA-tag was introduced at the C-terminus of SseF. The mutant alleles of *sseF* present on low copy number plasmids were analyzed in the background of the *sseF* strain. The *sseF* mutant strain complemented with a plasmid for the expression of WT *sseF* showed characteristics of *Salmonella* WT. Since all deletion constructs were expressed *in vitro* (not shown), we next examined if the SseF deletion variants were translocated into the host cell. All SseF variants were detectable and exhibited the same subcellular localization as WT SseF-HA (Fig. 1C). We quantified the signal intensities for immuno-staining of translocated SseF-HA and *Salmonella* LPS as a measure of the amount of intracellular bacteria. There was considerable variation between individual infected host cells at 16 h after infection. The average ratio of HA signals to *Salmonella* LPS signals was 4.1 for WT SseF, and ratios of 3.0, 3.2, 6.2, 5.3 and 3.1 were determined for SseF_Δ179–189_-HA, SseF_Δ195–200_-HA, SseF_Δ195–205_-HA, SseF_Δ200–205_-HA, and SseF_Δ206–212_-HA, respectively. Reduced ratios of 2.1 and 1.4 were recorded for SseF_Δ201–212_-HA and SseF_Δ201–212_-HA, respectively. These data indicate that deletions of domains in SseF have no major effect on the translocation and/or stability of the mutant forms of SseF.

Previous work showed that SseF plays a major role in the intracellular replication in HeLa cells [17]. We examined the effect of the various deletions on intracellular replication (Fig. 1B). Strain *sseF* [*sseF*_Δ206–212_] showed a replication rate comparable to that of the wild type. All the other mutants showed a replication defect comparable to that of *ssaV* or *sseF* mutant strains. The deletion of only 6 aa (SseF_Δ200–205_) was sufficient to inhibit the intracellular replication in HeLa cells.

In addition to the reduced intracellular replication, our previous work showed that strains deficient in *sseF* or *sseG* exhibit aberrant phenotypes with respect to the induction of SIF. The discontinuous endosomal aggregations induced by *sseF* or *sseG* strains were termed pseudo-SIF [17]. The typical structures of SIF and pseudo-SIF in infected and PFA-fixed cells are shown in Fig. 1D. To test the contribution of domains in SseF to induction of endosomal aggregates, HeLa cells were infected with strains expressing various *sseF* alleles and scored for SIF or pseudo-SIF phenotypes. We always found an inverse correlation between the numbers of cells showing SIF or pseudo-SIF phenotypes. The deletion of aa 206–212 did not impair the induction of SIF, whereas deletion of aa 201–212 resulted in a considerably reduced number of cells with SIF, comparable to the *sseF* strain. Deletions of aa 190–200, aa 195–205 and aa 200–205 also led to reduced SIF formation. The strains expressing *sseF*_Δ179–189_ or *sseF*_Δ195–200_ induced an intermediate phenotype with half of the cells showing SIF and the other half showing pseudo-SIF formation. The smallest deletion leading to markedly reduced formation of SIF was again the deletion of aa 200–205 (Fig. 1E). This mutation was also strongly attenuated in HeLa cells.

**Figure 2:**
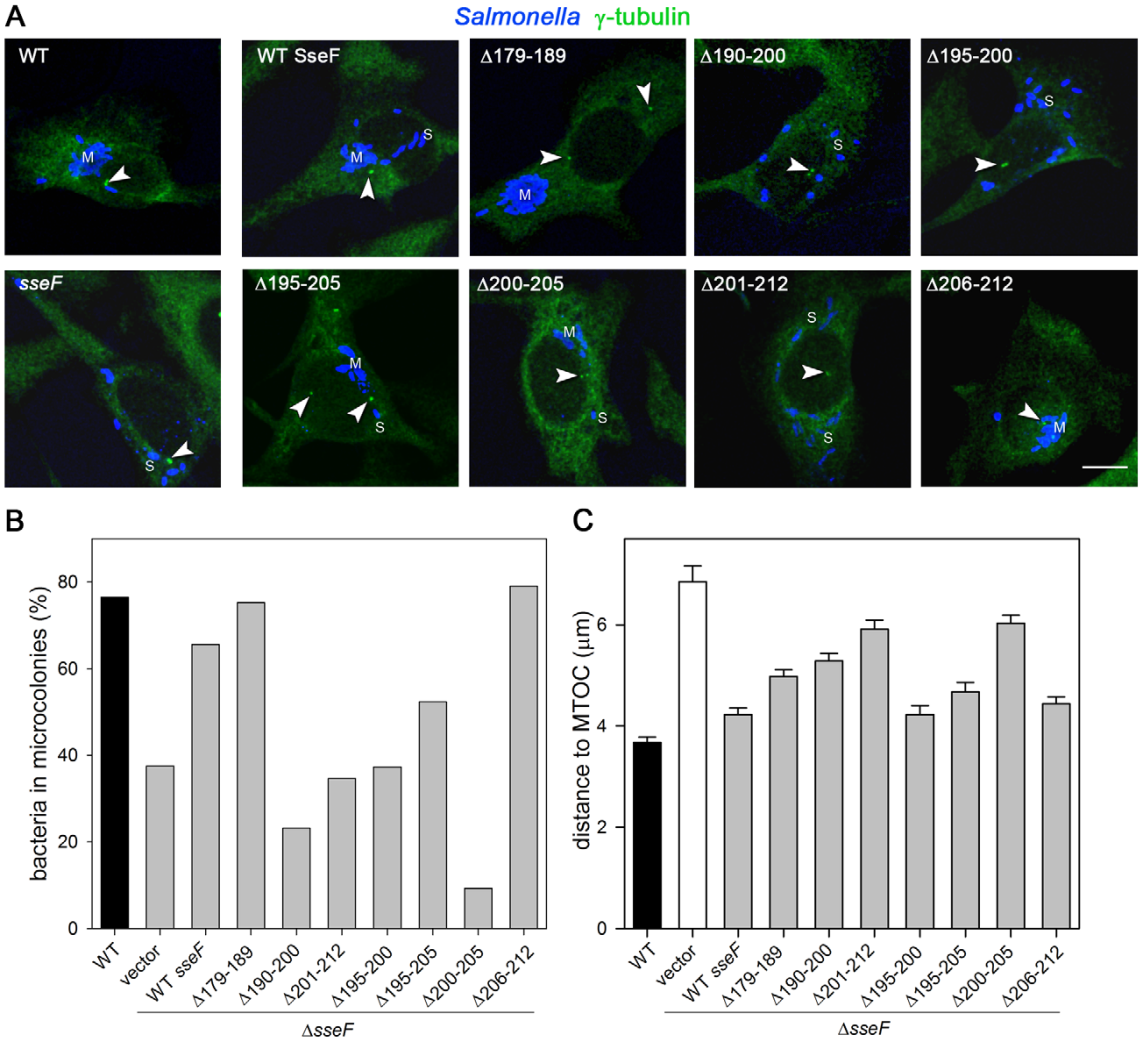
Role of the C-terminal hydrophobic domain in SseF for positioning of *Salmonella*-containing vacuoles in infected cells. HeLa cells were infected with *Salmonella* WT, the *sseF*-deficient strain or the *sseF*-deficient strain harboring plasmids for the expression of WT *sseF* or various mutant alleles. Cells were fixed with MeOH 16 h after infection and subjected to immuno-staining for the microtubule-organizing center (MTOC) using γ-tubulin antisera (detected with α mouse Alexa488, green), and for *Salmonella* using α O-antigen antisera (detected with α rabbit Alexa647). A) Representative infected cells are shown and arrowheads indicate the location of MTOC. In cells with multiple MTOC, the distance of the SCV to the proximal MTOC was determined. Microcolonies were defined as clusters of at least 5 bacteria in close proximity and examples are indicated by M. Scattered SCV are indicated by S. Scale bars, 20 µm. B) Intracellular *Salmonella* were scored for location in microcolonies or scattered SCV. At least 25 infected cells of approximately uniform size were identified, images were acquired using Leica SP5 CLSM and the percentage of bacteria in microcolonies was calculated. C) The distance between individual intracellular bacteria and the MTOC was determined using ImageJ software. Means and standard errors of mean for 250 to 600 intracellular bacteria per strains are shown and the data are representative for two independent experiments.

As a third approach to investigate the phenotype of deletions in *sseF*, we investigated the subcellular positioning of SCV as function of SseF. Previous work demonstrated that mutations in SseF result in formation of scattered SCV with peripheral localization, rather than microcolonies with juxtanuclear positions as observed for the WT strain [18], [19], [20]. As an indicator for the subcellular position of the SCV, the distance to the microtubule-organizing center (MTOC) was determined. We analyzed the microcolony formation and SCV-to-MTOC distance of WT, the *sseF* strain and the *sseF* strain expressing WT *sseF* or various mutant alleles (Fig. 2).

Next, the frequency of bacteria in microcolonies was determined (Fig. 2B). Deletions *sseF*_Δ179–200_ or *sseF*_Δ206–212_ had no effect on microcolony formation. Strains expressing *sseF*_Δ190–200_, *sseF*_Δ201–212_ or *sseF*_Δ195–200_ were as reduced in microcolony formation as the *sseF* strain. The strongest reduction in microcolony formation was observed for the strain expressing *sseF*_Δ200–205_, while the strain expressing *sseF*_Δ195–205_ exhibited intermediate characteristics.

The distance of individual intracellular bacteria to the MTOC showed considerable variation regardless of the *sseF* allele expressed. However, the quantification of SCV-to-MTOC distances of 250 to 600 individual SCV revealed specific characteristics. As observed before, the average distance of SCV containing the *sseF* strain to the MTOC was much higher than that of WT bacteria. Distances of *sseF* strains expressing *sseF*_Δ195–200_, or *sseF*_Δ206–212_ were similar to that of the WT strain. Strains expressing *sseF*_Δ719–189_, *sseF*_Δ190–200_, or *sseF*_Δ195–205_ formed SCV with increased distances to the MTOC. SCV with strains expressing *sseF*_Δ201–212_ or *sseF*_Δ200–205_ had highest average SCV-to-MTOC distances, although lower than that of SCV harboring the *sseF* strain.

In conclusion, analyses of intracellular replication, SIF or pseudo-SIF formation and positioning of the SCV indicated that the region of aa 200–205 in SseF comprising the motif AIGAVL has an apparently important role for the effector functions of SseF.

### Mutational analysis of the AIGAVL motif of SseF

**Figure 3:**
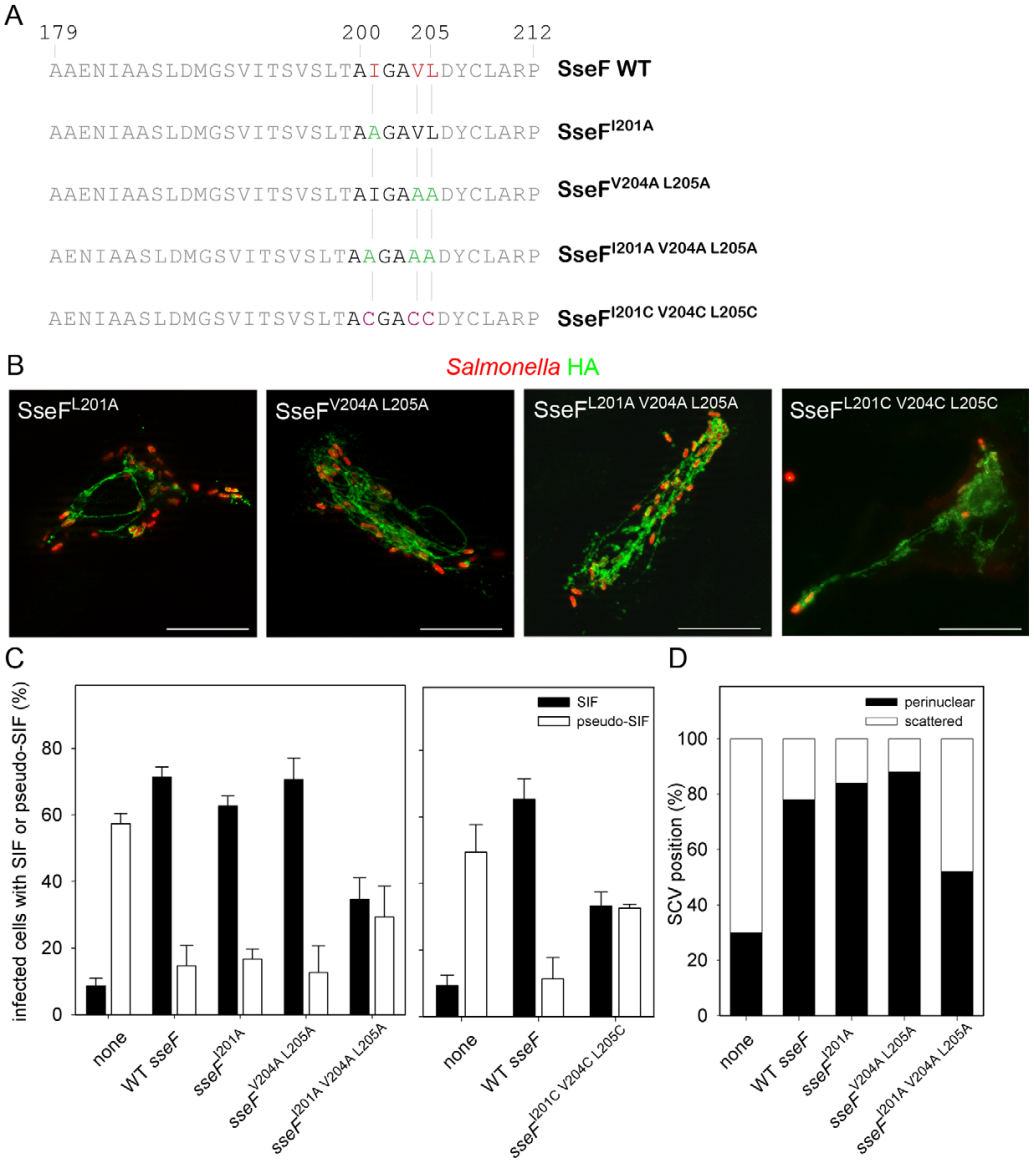
Site-directed mutagenesis of the AIGAVL motif of SseF. A) Selected hydrophobic aa residues in the AIGAVL motif were replaced by alanine or cysteine as indicated. All mutant alleles were expressed similar to WT *sseF* as determined by Western blot analyses (data not shown). B) The translocation of various mutant variants of SseF by intracellular bacteria was analyzed. HeLa cells were infected with the *sseF* strain harboring plasmids for expression of various alleles of *sseF*. Cells were fixed 16 h after infection and immuno-stained for the HA tag (green) and *Salmonella* LPS (red). Representative infected cells are shown. Scale bar: 20 µm. C) To quantify the effect of aa exchanges on SseF function, HeLa cells were infected with the *sseF* strain, or the *sseF* strain harboring plasmid for the expression *sseF*::HA or various *sseF* alleles as indicated. Cells were immuno-stained for LAMP2 and LPS, and scored for the formation of SIF (filled bars) and pseudo-SIF (open bars). In each experiment, at least 50 infected cells per condition were counted and means and standard deviations of three individual experiments are shown. D) The subcellular localization of SCV harboring *sseF* strains expressing various *sseF* alleles was analyzed. Infected cells were scored for formation of perinuclear microcolonies (black bars) or scattered SCV (open bars).

The AIGAVL motif is not related to any motif with known function in other proteins. In order to elucidate if individual aa within the motif are required for the effector function, or if the hydrophobicity of the residues alone is sufficient to maintain the effector function, different aa exchanges were performed within the AIGAVL motif. Hydrophobic residues present within the motif were exchanged against alanine (Fig. 3A).

All SseF variants with exchanges of hydrophobic aa were synthesized *in vitro* (not shown). To examine if the protein variants are still translocated into the cytoplasm of host cells, HeLa cells were infected with strains expressing the mutant *sseF* alleles. Following infection, strong signals for the HA-tag were detectable and the subcellular distribution of SseF variants was comparable to that of WT SseF (Fig. 3B). Image analyzed war performed as for the various deletion variants (Fig. 1) and resulted in HA-to-bacteria ratios of 1.9, 3.3, 1.4, and 1.8 for SseF^I201A^-HA, SseF^V204A^ ^L205A^-HA, SseF^I201A V204A L205A^-HA, and SseF^I201C V204C L205C^-HA, respectively. We next analyzed the phenotypes of these SseF variants. After infection with an *sseF* strain translocating SseF^I201A^, the amounts of infected cells showing SIF formation was comparable to WT-infected cells, indicating the this exchange did not affect SIF formation (Fig. 3C). The same observation was made for the exchange of the residues SseF^V204A^ ^L205A^. However, the exchange of all three hydrophobic aa against alanine resulted in an intermediate phenotype with approximately equal numbers of cells positive for SIF and pseudo-SIF (Fig. 3C). We also scored the effect of mutations in AIGAVL motif on the subcellular localization of the SCV (Fig. 3D). While SCV harboring the *sseF* strain most frequently showed a scattered distribution, strains translocating WT SseF, SseF^I201A^ or SseF^V204A^ ^L205A^ were predominantly localized in SCV with a perinuclear position. The strain translocating SseF^I201A V204A^ ^L205A^ showed an intermediate phenotype. Overall, analyses for the various SseF variants with respect to SIF or pseudo-SIF formation and subcellular location of the SCV were in close correlation.

In order to analyze if the polarity of the aa within the AIGAVL motif sequence is important, all non-polar hydrophobic amino acids present in the motif were replaced against arginine (R), a basic aa. The resulting constructs were tested for the expression *in vitro* (not shown). The mutant protein was not detectable by Western blot analyses, indicating that the exchange against arginine led to highly reduced synthesis or stability of the protein. It is conceivable that this mutation dramatically affects the secondary structure of the protein. We next replaced non-polar hydrophobic aa in AIGAVL by cysteine as a polar, less hydrophobic aa. The substitution to ACGACC did not impair the translocation of the protein (Fig. 3B). The subcellular localization of SseF^I201C V204C L205C^ was comparable to that observed for WT SseF (data not shown). An intermediate level of SIF and pseudo-SIF formation was also observed for this mutant and the phenotype was comparable to that induced by strains translocating SseF^I201A V204A L205A^.

**Figure 4:**
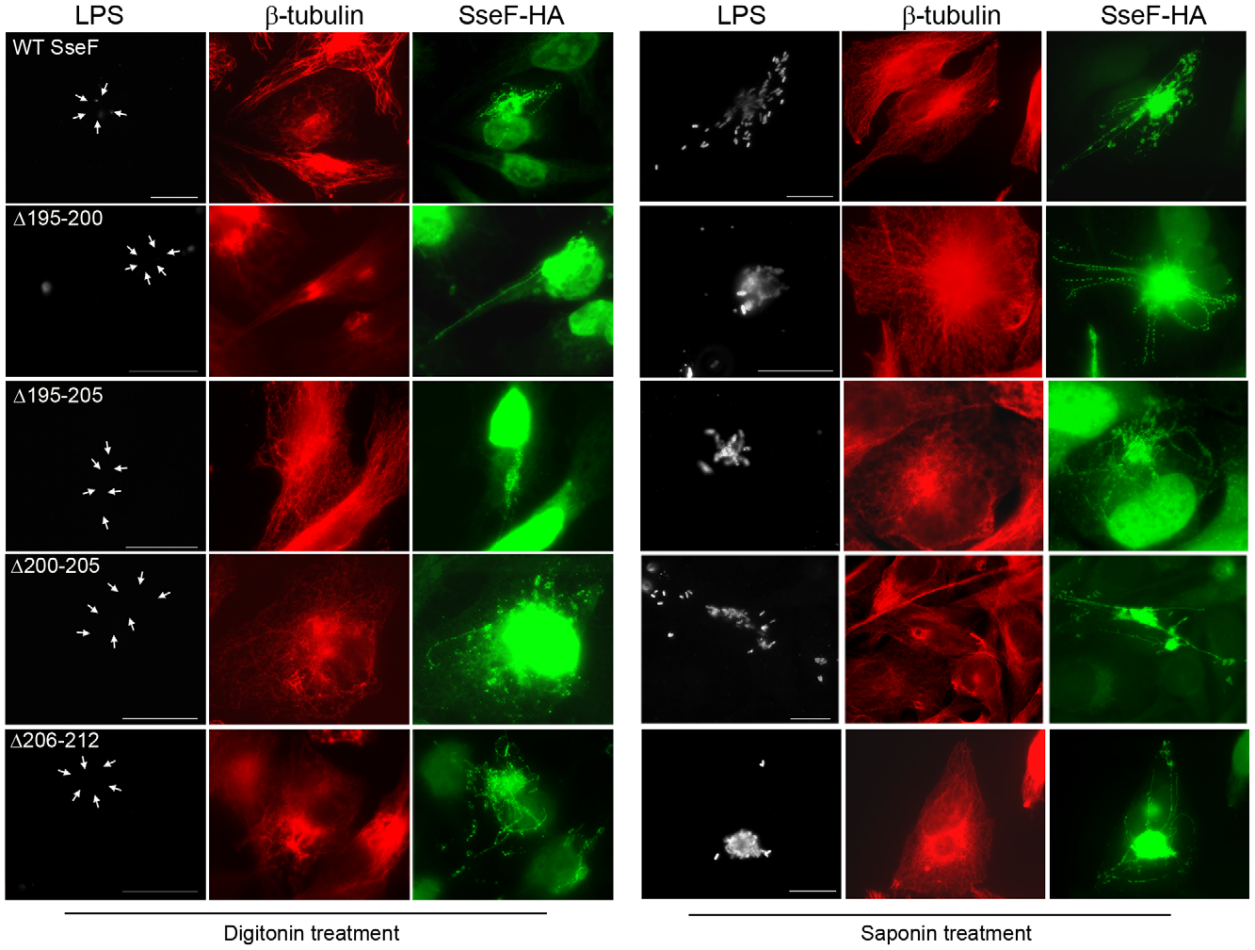
Membrane topology of translocated SseF. HeLa cells were infected with the *sseF* strain harboring plasmids for the expression of HA-tagged alleles of *sseF*. At 16 h after infection, the cells were fixed and subjected to permeabilization by saponin or digitonin as indicated. Saponin treatment allows the penetration of antibody across the cytoplasmic and endosomal membranes of the host cell, while digitonin treatment only permeabilizes the cytoplasmic membrane. To control the selectivity of the permeabilization, intracellular *Salmonella* were immune-stained with antibody against LPS (white). Detection of translocated SseF was performed with antibody against the HA tag (green). As control for a cytosolic protein, host cell β-tubulin was labeled (red). In order to locate intracellular bacteria, DAPI staining of bacterial DNA was performed and the location of DAPI-stained bacteria is indicated by arrows for the digitonin experiment (DAPI staining not shown).

To control if the deletions described above affect the localization of SseF, we investigated the topology of various SseF variants after translocation. Using differential permeabilization, we previously found that the C-terminus of membrane-associated SseF is exposed to the cytoplasmic face of endosomal membranes [19]. Differential permeabilization was performed with cells infected with strains expressing WT *sseF* or various mutant alleles. As shown in Fig. 1, Fig. 2, and Fig. 3, the deletion of only 6 amino acids had a dramatic effect on the effector functions of SseF. One reason for this loss of function could be that the deletion of these amino acids resulted in a conformational change in the protein structure. The C-terminal region of SseF, facing the host cell cytoplasm, might be required for the interaction with other effectors or host cell proteins. By conformational changes due to deletions within SseF, the C-terminus would no longer be accessible for putative interaction partners. To control if the C-terminus of these deletion variants is still directed to the cytoplasmic face and not facing the SCV lumen, HeLa cells infected with various strains were selectively permeabilized with digitonin and immuno-stained for the HA-tag 16 h after infection. HeLa cells permeabilized with saponin served as control. Translocation of all deletion variants was detectable in digitonin-permeabilized cells (Fig. 4). The C-terminal HA-tag was accessible, indicating that the C-terminus is still directed to the cytoplasmic face. This result is an indication that no conformational change affecting the position of the C-terminal part of SseF was induced by these deletions. However, this experimental approach cannot elucidate if deletions affect topology of the AIGAVL motif and its accessibility for putative interacting proteins. We also inserted HA-tags in various positions of SseF, but these variants were not detectable by immune-fluorescence (data not shown).

### SseF and SseG can act locally or on distance

**Figure 5:**
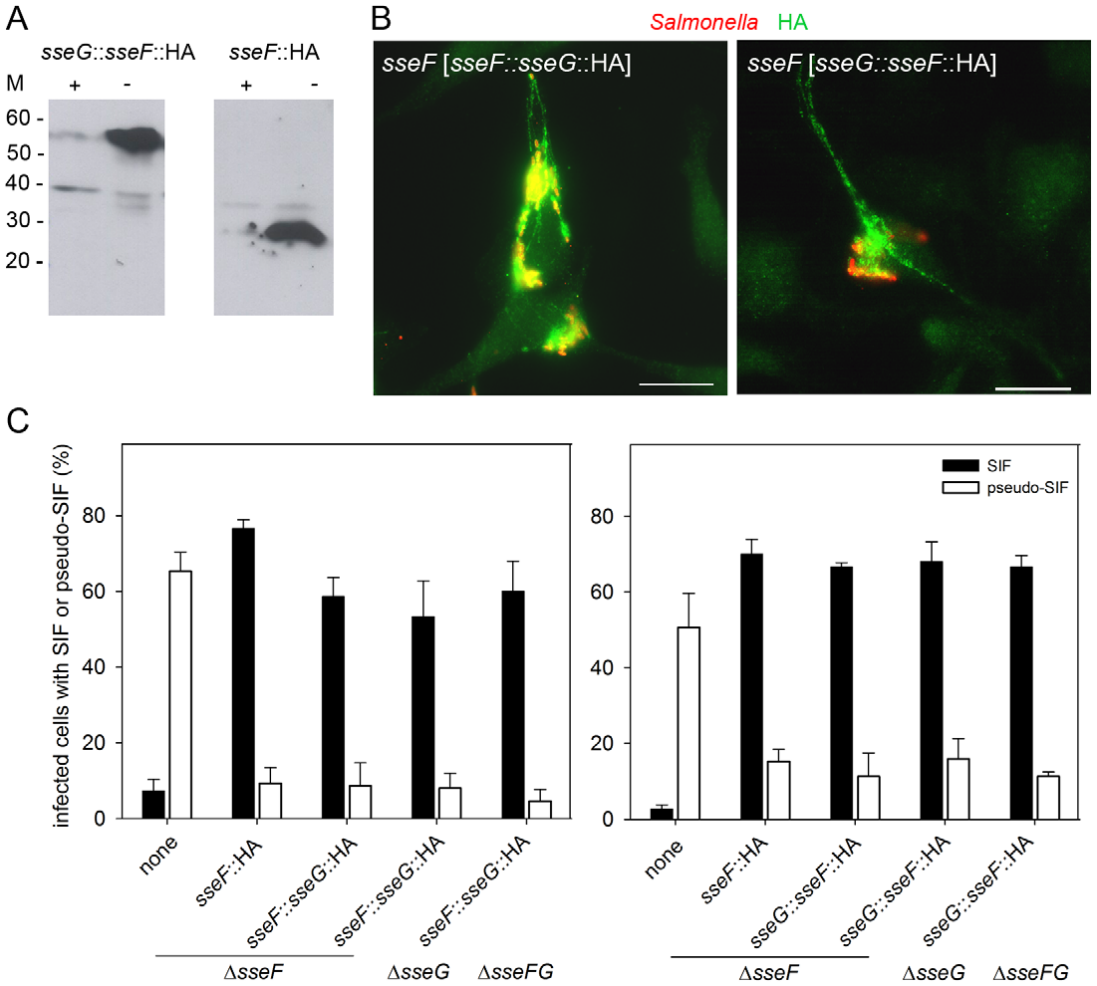
SseFSseG or SseGSseF fusion proteins can functionally replace SseF and SseG and contribute to SIF induction. A) The *sseF* strain harboring plasmids for the expression of *sseF*::HA or *sseG*::*sseF*::HA was grown over night under SPI2-inducing conditions (PCN medium with 0.4 mM Pi, pH 7.4, indicated by +), or under non-inducing conditions (PCN medium with 25 mM Pi, pH 7.4, indicated by −). Equal amounts of bacterial cells were harvested and lysed, protein was separated by SDS-PAGE and the HA tag detected by Western blot analysis. The theoretical molecular mass of SseF-HA and SseG-SseF-HA is 27 kDa and 52 kDa, respectively. B) Translocation of SseF-SseG-HA and SseG-SseF-HA. HeLa cells were infected at a MOI of 10, fixed 16 h p.i. and immuno-stained for HA-tag (green) and for *Salmonella* LPS (red). Representative merge pictures are shown. Scale bar: 20 µm. C) Function of SseF-SseG-HA (left panel) and SseG-SseF-HA (right panel) fusion proteins in SIF formation. HeLa cells were infected with *sseF*, *sseG* or *sseFG* strains harboring plasmids for expression of *sseF*::HA, *sseF*::*sseG*::HA or *sseG*::*sseF*::HA as indicated. Cells were fixed 16 h after infection and immuno-stained for LAMP2 and *Salmonella* LPS. For each condition, 50 infected cells were scored for the presence of SIF (filled bars) or pseudo-SIF (open bars). The means and standard deviations of three independent experiments are shown.

We observed that the effectors SseF and SseG frequently colocalize on endosomal membranes after translocation. Previous works also suggested that SseF and SseG interact in eukaryotic host cells [21]. Based on these observations, we questioned if functions of SseF and SseG are maintained if both effector proteins are translocated as fusion protein. Constructs were generated that expressed *sseFG*::HA or *sseGF*::HA gene fusions. Fusion proteins SseGF-HA (Fig. 5A) and SseFG-HA (data not shown) were synthesized by *Salmonella* strains grown under SPI2-inducing conditions, efficiently translocated by intracellular *Salmonella* and showed a subcellular localization similar to that of SseF or SseG (Fig. 5B). We next investigated the complementation of mutations in *sseF*, *sseG* or *sseFG* by *sseGF*::HA or *sseFG*::HA and scored the induction of SIF or pseudo-SIF in infected HeLa cells (Fig. 5C). The intracellular phenotype of various strains translocating SseFG or SseGF fusion proteins was identical that of the *sseF* [*sseF*] strain, i.e. SIF formation was comparable to that observed in WT-infected cells. The results indicate that the functions of SseF and SseG can be combined into a single polypeptide.

**Figure 6:**
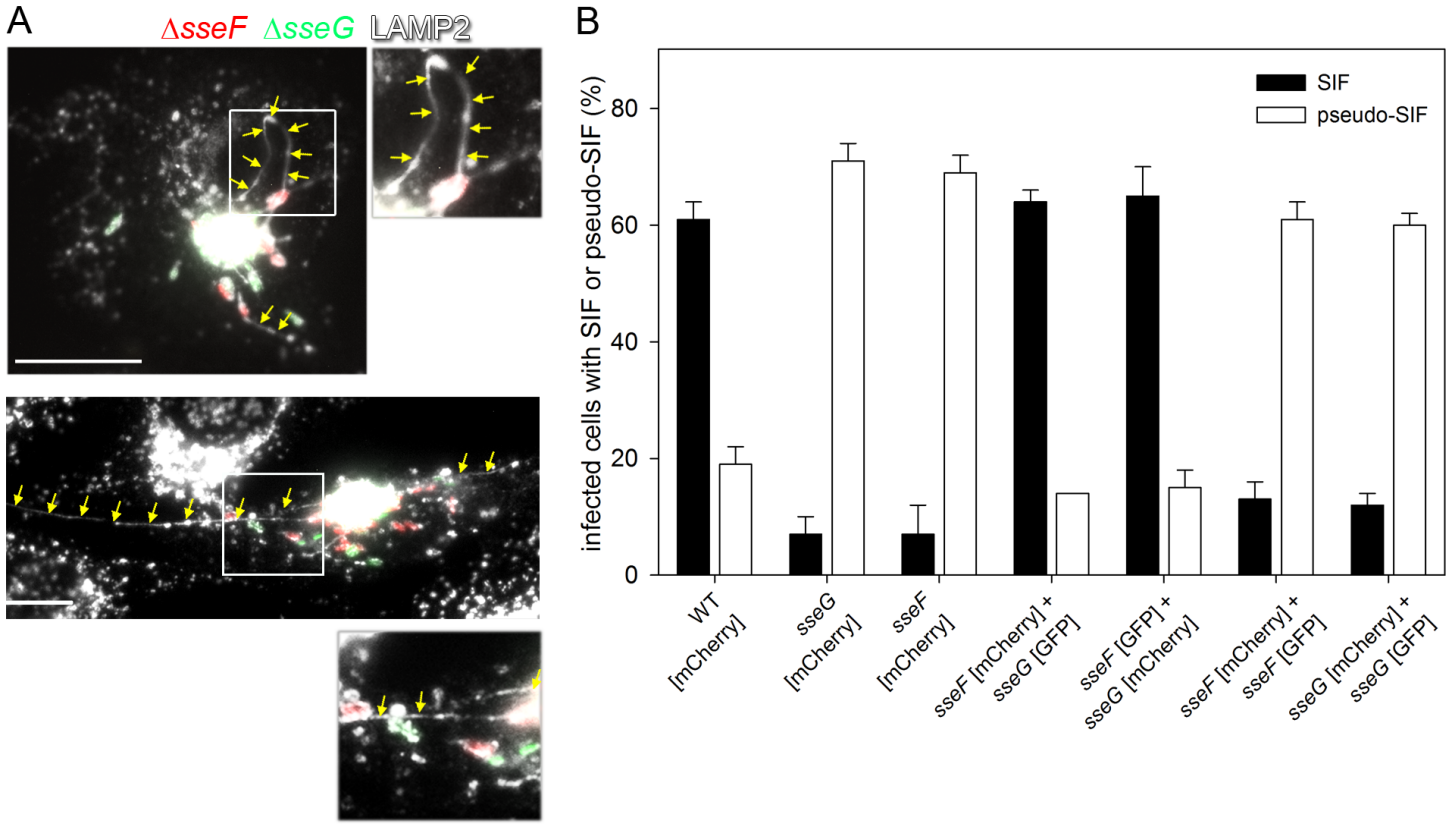
SseF and SseG function *in trans* in modifying the host cell endosomal system. HeLa cells were infected with individual *Salmonella* WT, *sseF* or *sseG* strains each harboring a plasmid for expression of mCherry. Further cells were co-infected with a mixture consisting of *sseF* and *sseG* strains expressing mCherry or GFP for distinction of the strains. As controls, co-infection with *sseF* strains expression mCherry or GFP or *sseG* strains expressing mCherry or GFP was performed. In order to obtain sufficient numbers of co-infected cells, an MOI of 100 was used. A) SIF formation in representative cells co-infected with *sseF* [mCherry] (red) and *sseG* [GFP] (green). The cells were fixed 16 h p. i. and immuno-stained for LAMP2 (shown in white). Arrows indicate continuous SIF in co-infected cells. Scale bar: 20 µm. B) HeLa cells infected with single strains or co-infected with strains were identified and scored for formation of SIF or pseudo-SIF. At least 50 cells per condition were scored and the mean percentages and standard deviations of three independent experiments were calculated for infected/co-infected cells showing SIF (filled bars) or pseudo-SIF (open bars).

**Figure 7:**
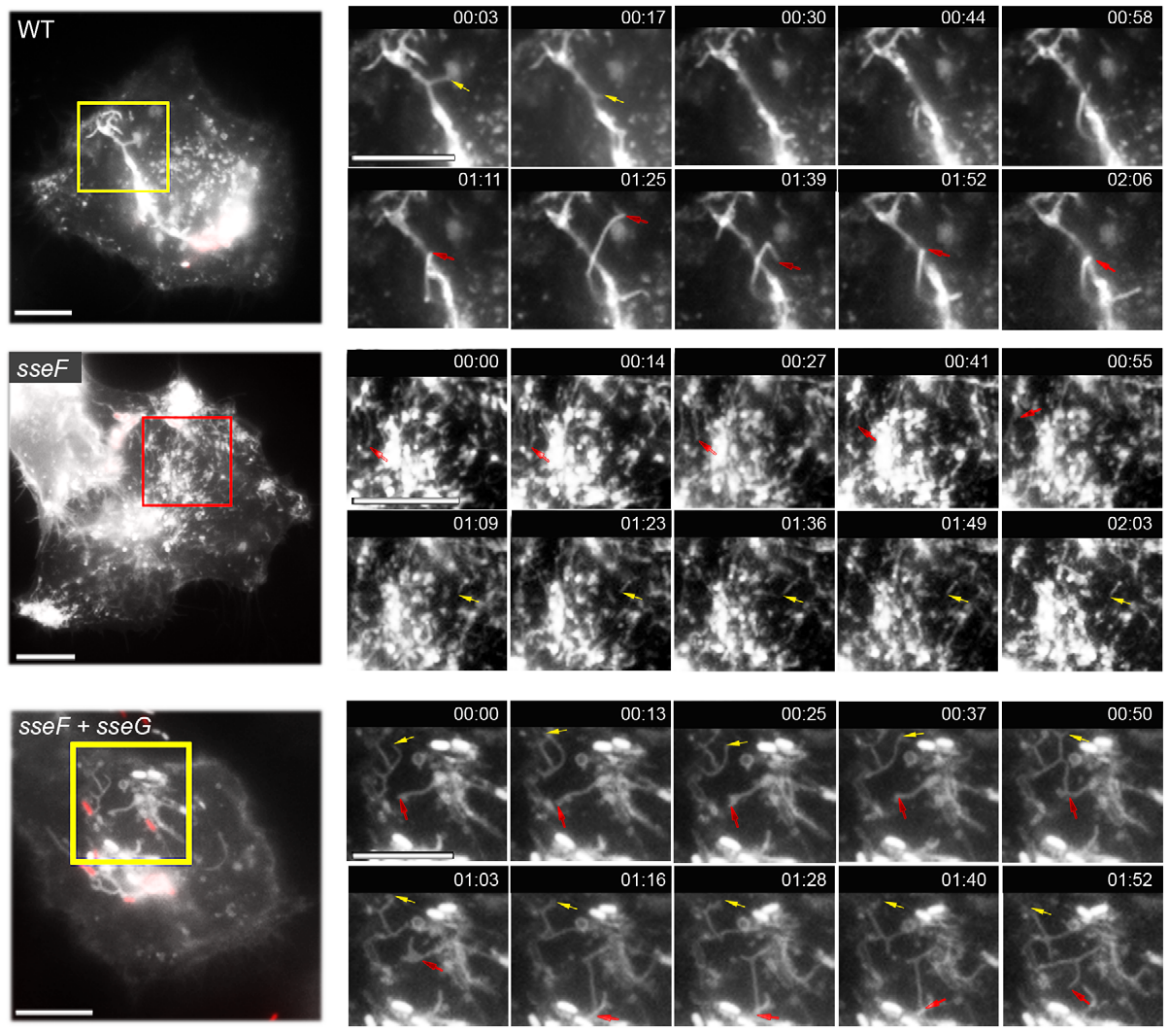
SIF formation in living cells after co-infection with *sseF* and *sseG* strains. HeLa cells were transfected with a plasmid for the expression of LAMP1-GFP and infected at an MOI of 50 with *Salmonella* WT [mCherry], *sseF* [mCherry], or co-infected with *sseF* [mCherry] and *sseG* [GFP]. Live cell imaging was performed 5 h p.i. for a period of 5 min. using a Zeiss Axiovert 200 M wide field microscope equipped with an environmental chamber. The rectangles in overview images indicate detail sections shown as time series. The acquisition time is indicated in min∶sec. Green fluorescence (LAMP1-GFP and *sseG* [GFP]) is shown in white and arrows indicate the extension and contraction of SIF. Note the appearance of extended continuous SIF in WT and *sseF*/*sseG* co-infected cells and the fine and short tubular structures that appear in the cell infected with the *sseF* strain. Scale bar: 10 µm.

In a second experiment, we tested if the translocation of SseF and SseG by distinct bacteria present in the same host cell would restore SIF formation. To test this experimentally, cells were either infected with single strains, or co-infected with the *sseF* strain labeled with mCherry and the *sseG* strain labeled with GFP, and scored for SIF and pseudo-SIF formation (Fig. 6). Co-infection with *sseF* [mCherry] and *sseG* [GFP] led to similar numbers of cells with SIF as WT-infected cells, whereas infection with *sseF* [mCherry] or *sseG* [GFP] alone resulted in highly reduced SIF formation. Representative cells are shown in Fig. 6A. In addition, we analyzed if co-infection with *sseF* [mCherry] and *sseF* [mCherry], or *sseG* [mCherry] and *sseG* [GFP] restored SIF formation. As expected, the cells showed increased appearance of pseudo-SIF reaching the level of HeLa cells infected with single mutant strains (Fig. 6B). The presence of SseF and SseG, even if translocated by different bacteria, was sufficient to induce SIF formation. These experiments could not clarify if the spatial proximity of translocated SseF and SseG within the cell is required for the induction of SIF, because in most of the double infected cells the two strains were located in the same, usually perinuclear area. In addition, SIF formation was analyzed in living cells after co-infection with *sseF* and *sseG* strains (Fig. 7). For this aim, HeLa cells were transfected with a plasmid for the expression of LAMP1-GFP. These cells were either infected with WT [mCherry], *sseF* [mCherry], or co-infected with *sseF* [mCherry] and *sseG* [GFP]. Infection with *Salmonella* WT resulted in a highly dynamic network of tubular structures as previously observed [8], [9]. Infection with *sseF* [mCherry] also resulted in tubular membrane structures with a much thinner and shorter appearance. These structures did not grow and collapse as SIF observed after infection with *Salmonella* WT. Co-infection with *sseF* [mCherry] and *sseG* [GFP] resulted in tubular structures strongly resembling SIF formation induced by WT infection and these filaments appeared also highly dynamic.

### SseF is an integral membrane protein after translocation into host cells

In a previous study we reported that SseF is predicted as a membrane protein with two extended hydrophobic regions [19]. The bioinformatics analysis (TMpred) [22] predicted 4–5 transmembrane domains [19]. After subcellular fractionation of infected RAW264.7 cells, SseF was found in the membrane fraction [10]. The prediction of membrane-spanning domains and the observations that SseF was co-localized with LAMP1-positive vesicles in HeLa cells and was found in the membrane fraction in RAW264.7 cells, raised the question how translocated SseF is associated with host cell membranes.

**Figure 8:**
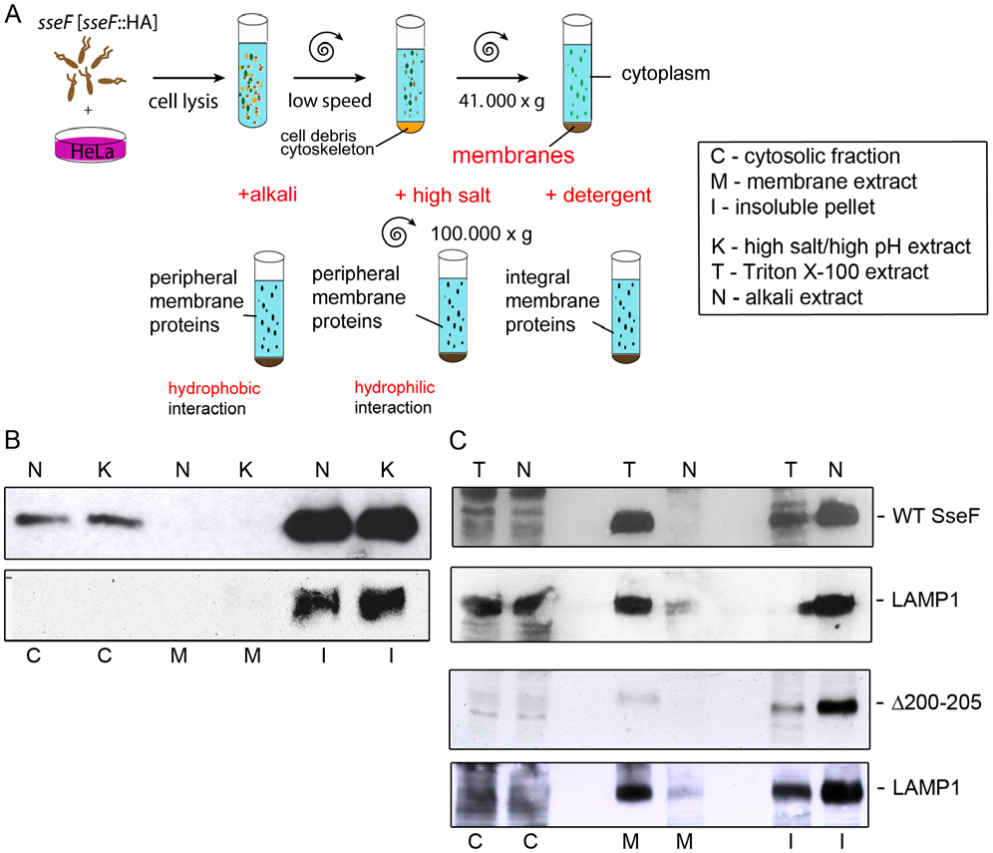
Translocated SseF is an integral membrane protein. A) Schematic representation of the procedure used for the analysis. HeLa cells were infected at an MOI of 10 with the *sseF* strain harboring a plasmid for the expression of *sseF*::HA (WT SseF) or *sseF*_Δ200–205_ (Δ200–205) as indicated. Cells were harvested 16 h p.i. and subjected to subcellular fractionation after mechanical lysis. The membrane fraction was used for extraction under conditions of different stringency. The cytoplasmic fraction (C) was separated from the membrane pellet as described in the Experimental Procedures. B) The membrane fraction was incubated with high salt/high pH buffer (K) or with alkaline buffer (N) in order to extract peripheral membrane proteins with hydrophobic or hydrophilic interaction with membranes, respectively. C) The membrane fraction was treated with Triton X-100 buffer (T) to extract integral membrane proteins, and with alkaline buffer (N) as control. C indicates the cytosolic fraction, M the membrane extract and I the insoluble membrane pellet. The various fractions were subjected to SDS-PAGE on 12% gels and the proteins were transferred onto nitrocellulose membranes. The HA epitope tag was detected using antibodies against the HA-tag and as controls, the Western blots were incubated with antibodies against LAMP1 as an integral protein of endosomal membranes.

To characterize the association of SseF with host cell membranes, HeLa cells infected with the *sseF* strain expressing *sseF*::HA were subjected to subcellular fractionation and the membrane-containing pellets were subsequently extracted with a detergent-containing buffer, a high salt/high pH buffer, or a high alkaline buffer. With the high salt buffer extraction, peripheral membrane proteins associated by hydrophilic interactions can be extracted. Proteins associated by hydrophobic interaction can be resolved with alkaline buffer and treatment with the detergent-containing buffer allows extraction of all classes of membrane proteins which includes also integral membrane proteins (Fig. 8A).

As shown in Fig. 8B, SseF-HA could only be detected in the insoluble fraction after extraction with high salt/high pH buffer or alkaline buffer. However, SseF-HA was not detectable in the soluble fraction. There was only a small portion of protein detectable in the cytoplasmic fraction. The signal for SseF-HA appeared in the same fraction as the integral host cell membrane protein LAMP1. This indicates that SseF is not a peripheral membrane protein associated by hydrophilic or hydrophobic interactions. To test if translocated SseF is integrated into host cell membranes, membranes of HeLa cells infected with *sseF* [*sseF*::HA] were extracted with Triton X-100-containing buffer (Fig. 8C). After treatment of the membrane fraction with Triton X-100 buffer the signal for SseF-HA appeared in the soluble membrane fraction (M), whereas after alkali extraction the signal remained in the unsolvable membrane fraction (I). Again, SseF-HA was detectable in the same fractions as the integral membrane protein LAMP1. We also investigated the localization of SseF_Δ200–205_ and found that this mutant form of SseF exhibits the same characteristics as WT SseF with respect to the solubilization by detergent-containing extraction buffer. From these experiments, we concluded that SseF-HA has the characteristics of an integral membrane protein after its translocation.

## Discussion

In this study, we demonstrate that a short hydrophobic sequence motif is essential for the effector function of SseF after translocation by the SPI2-T3SS. To our knowledge, this motif with the sequence AIGAVL is neither present in other T3SS effector proteins nor known as a functional motif in mammalian host cells proteins.

Effector proteins of T3SS have been reported to adopt rather different subcellular localizations after translocation into host cells. A large group of effectors shows a rather homogenous distribution in the cytoplasm, while other effectors target the nucleus of the host cell or interact with membranes of organelles. A specific characteristic of a subset of effector proteins of the SPI2-T3SS is their close association with the late endosomal/lysosomal membrane system. While some of these effectors are characterized by the STE motif [23], the highly hydrophobic character of SseF has been considered as a main factor for association with host endosomal membranes [17]. The targeting of SseF to endosomal membranes reported before [17] and the membrane integral nature of the protein reported here raises the question how a bacterial effector can be targeted to and inserted into host cell membranes. Other effectors such as SifA contain specific motifs for modification by prenylation, but no such motif was identified in SseF. Membrane insertion of host cell proteins require the contribution of chaperones such the Hsp70 family for insertion in the mitochondrial membrane, or the protein translocator for insertion into ER membranes. A specific chaperone of SseF is SscB, which binds to SseF in the bacterial cytoplasm prior to translocation. Dedicated chaperones of T3SS effectors are not known to be co-translocated with the effector and we did not observe that this dedicated chaperone is translocated into host cells (data not shown). We speculate that SseF and possibly also SseG and other membrane-associated SPI2-T3SS effectors either deploy host cell chaperones in order to achieve membrane-integral localization, or that the transfer from the host cell cytoplasm to endosomal membranes is solely determined by the sequence of SseF without the support by host cell factors.

SseF and SseG are specific effector proteins of *S. enterica*. SseF and SseG are considered as specific virulence proteins of *S. enterica* and no homologues have been identified so far. However, recent analyses identified pathogenicity islands that encode T3SS with significant similarity to the SPI2-T3SS. Interestingly, the PAI of *Edwardsiella tarda* [24] encodes proteins with partial similarity to SseF and SseG and genes with similarity to *sseF* were identified in the genomes of environmental *Yersinia* species and *Shewanella baltica*. A PAI with similarity to SPI2 has also been identified in *Chromobacterium violaceum* [25]. Due to the limited understanding of the pathogenesis of these bacteria and their interaction with host cells, there is also lack of knowledge if effector proteins of *Edwardsiella* or *Chromobacterium* fulfill similar functions.

Mutants defective in either SseF or SseG, or both proteins show similar phenotypes with respect to the formation of SIF, positioning of the SCV or the intracellular replication of *Salmonella* ([17], [19], this study). Thus, SseF and SseG may have partially redundant functions. SseF was shown to be permissive to the C-terminal fusion of various tags of heterologous proteins and was used for the delivery of recombinant antigens by *Salmonella* vaccine carriers [26]. SseF and SseG are functional if translocated as SseFG or SseGF fusion proteins. This result indicates that the functional parts of both proteins can be joined to a single polypeptide and affect host cell functions from the same subcellular localization after translocation. Previous work reported the interaction of both effectors after translocation or co-expression from transfection vectors [21]. We and others observed that SseF, SseG and other effector proteins are closely colocalized with endosomal membranes after translocation and are distributed through the cell with the extension of SIF [17], [27].

A major challenge for future work will be the identification of the host cell interaction partner for SseF and SseG. The highly hydrophobic nature of both proteins has restricted previous approaches for screening of protein interactions as well as genetic screens such as yeast two hybrid assays. However, a recent study showed that *sseF* can be expressed in yeast, resulting in an inhibition of growth and alterations of the actin cytoskeleton [28].

During a screen for the biological activity of *Salmonella* effector proteins *in planta*, the Börnke group observed that SseF specifically induced a hypersensitive response (HR) in tobacco [29]. This work used an *Agrobacterium* delivery system for the transfer of an *sseF* expressing vector into tobacco cells. This resulted in a HR response that showed all signs of immune reactions induced by effector proteins of T3SS of plant-pathogenic bacteria. Interestingly, mutant forms of SseF with deletions of aa 179–212 or more specifically the AIGAVL motif (aa 200–205) were defective in induction of the HR. The results were further confirmed by T3SS–dependent translocation of an AvrA-SseF fusion by *Xanthomonas campestris*. The subcellular localization of SseF in plant cell could not be analyzed due to the induction of cell death, however, the functionally inactive form of SseF with deletion of aa 200–205 showed a remarkable association with the endoplasmic reticulum (ER). This subcellular localization has not been observed in mammalian cells and SseF was rather found associated with endosomal membranes and microtubules. Since ER tubules are guided by the microtubule cytoskeleton, it is conceivable that the microtubule association of SseF induces a preferential interaction with ER membranes in plant cells.

The host cell interaction partners of SseF and SseG are still elusive and the hydrophobic properties of both effectors complicate screening approaches to identify such targets. We found that a short hydrophobic motif in SseF is essential for the effector function. The deletion of the motif had no detectable effect on the subcellular localization or topology of the mutant protein and the hydrophobic vicinity of the AIGAVL might indicate that this region is membrane integral. These findings suggest that host cell interaction partners might also be membrane-integral or membrane-associated proteins of endosomes. The interaction of SseF and SseG with these targets contributes to the fusion of endosomal membranes and formation of the extensive SIF network. Previous work demonstrated the role of microtubule motor proteins in intracellular replication of *Salmonella* and modification of the endosomal system of infected cells. The presence of membrane-integral SPI2 effector proteins could affect the interaction of motor proteins with vesicles and by this enable fusion events that otherwise would not occur.

SseF and SseG interact with each other after translocation into host cells. The distribution of both effectors appears rather similar after translocation and both proteins colocalize in a prominent manner with endosomal markers. Despite the similar distribution, distinct difference in the localization of SseF and SseG have been observed [10], suggesting that both effectors may have slightly different specificities. The differences in subcellular localization and the fact that SseF and SseG interact would allow a more simple explanation for their function in endosomal fusion. Interaction of SseF and SseG present in distinct subpopulation of endosomes directly mediates contact between vesicles and their fusion. In this model, it is not likely that the SseF/SseG interaction is directly mediated by membrane-integral parts of SseF, but the integrity of these portions could have subtle effects on the protein topology and the ability of a domain in SseF that is exposed to the cytoplasmic phase and required for interaction with SseG and thereby vesicle fusion. In this model, the presence of specific interaction partner of the host cell would not be required, and vesicle would be directly mediated by SseF-SseG interaction after insertion into the proper host cell membrane comportment. The model would also be in line with our observations that SseFSseG fusion proteins are functional as well as trans-complementation in cells co-infected with *sseF* and *sseG* strains. Such ‘simple’ functions are usually not considered for T3SS effectors. SseF and SseG belong to the few effectors encoded by genes within SPI2, while the majority of SPI2-T3SS effectors are encoded outside of SPI2. It is conceivable that SseF and SseG represent the evolutionary most ancient set of SPI2-T3SS effectors that was complemented by additional, more specialized effectors during evolution and adaptation to mammalian hosts. Fusogenic activity of SseF and SseG might have allowed a limited modulation of the host endosomal system sufficient to enable intracellular *Salmonella* to establish a foothold in host cells. The latter acquisition of effectors such as SifA or PipB2 with the dedicated host cells targets SKIP or kinesin, respectively, then let to an increased potential to alter host cell vesicular transport and to maintain the SCV during massive intracellular replication and to withstand otherwise highly effective immune defenses.

## Materials and Methods

### Bacterial strains and culture conditions

*Salmonella enterica* serovar Typhimurium strain 12023 was used as wild-type strain. The different bacterial strains used in this study are listed in Table 1 and the various plasmids used are described in Table S1. For cloning procedures, *Escherichia coli* strains DH5α and XL10 Gold were used.

**Table 1:** Strains used in this study.

strain	relevant properties	reference
*Salmonella* strains:		
NCTC12023	WT	lab collection (NCTC, Colindale, UK)
HH107	Δ*sseF*::*aphT*	[35]
HH108	Δ*sseG*::*aphT*	[35]
MvP373	Δ*sscB sseF sseG*	[10]
P2D6	*ssaV*::mTn*5*	[36]
*E. coli* strains:		
DH5α	general cloning strain	Invitrogen
XL10 Gold	general cloning strain	Stratagene
Phages:		
P22 HT	highly efficient transduction	[37]

Bacteria were grown in LB broth or on LB agar plates. If necessary for the maintenance of plasmids or selection of recombinant strains appropriate antibiotics (50 µg×ml^−1^ kanamycin or 50 µg×ml^−1^ carbenicillin) were added to broth or agar plates. For the experiments bacterial strains were freshly streaked on agar plates. Stock cultures were stored in 7% DMSO at −70°C.

### Construction of mutant alleles of sseF

All the deletion variants used in this study were plasmid based and brought into HH107 (*sseF*) background. In frame deletions were performed with a long range one-step PCR reaction with the oligonucleotides listed in Table S2 and p2643 as template. PCR products were digested with *Dpn*I (Fermentas) and purified by EtOH precipitation. The resulting pellets resuspended in H_2_O were directly used for electroporation. The constructs were confirmed by sequencing. For the generation of SseF_Δ206–212_ long range PCR was performed using high copy vector p3402 as template. After sequencing, the resulting plasmid was digested with *Hin*dIII and *Xba*I (Fermentas) and the insert subcloned into pWSK29.

### Construction of fusion proteins

The construction of SseGSseF was performed by SOE-PCR (splicing by overlap extension polymerase chain reaction). In the first round PCR reactions were performed with oligonucleotides SseG-For-*Eco*RI-2 and SseG-Rev (-Stop) with p2644 as template and oligonucleotides SseF-For (−Met) and SseF-Rev-HA-*Xba*I with p2643 as template. In a second round PCR the purified PCR products of the first round were mixed together and served as template with SseG-For-*Eco*RI-2 and HA-Rev-*Xba*I. The PCR reaction was gel purified, digested with *Eco*RI and *Xba*I and subcloned into p3351. The plasmid p3351 was generated by a PCR reaction using the primers ProB-For-*Kpn*I and SscB-Rev-*Eco*RI-2 together with p2643 as template. The resulting fragment were digested with *Kpn*I and *Eco*RI (Fermentas) and subcloned into pWSK29.

The generation of p3122 was also performed by SOE-PCR. A first round of PCR was performed using oligonucleotides SseGF-Fusion P2 and SseG-HA-Rev together with p2644 serving as template and SseGF-Fusion P3 and SseF-For *Eco*RI together with the template p2644. In the second PCR round, the PCR-products resulting from the first round were mixed together and served as template with SseF-For-*Eco*RI and SseG-HA-Rev. The gel-purified PCR product was digested with *Pst*I and *Xba*I (Fermentas) and subcloned into p2644.

### Cell culture and infection of HeLa cells

The human epithelial adenocarcicoma cell line HeLa was obtained from Cell Line Services (Heidelberg) and cultured in Dulbeco's modified Eagle medium (DMEM, PAA) containing 10% fetal calf serum, 4.5 g×l^−1^ glucose and 2 mM glutamine at 37°C in an atmosphere containing 5% CO_2_. For infection of HeLa cells, bacterial strains were grown in LB with appropriate antibiotics over-night, diluted in fresh medium and subcultured for 3.5 h to reach the late logarithmic phase. The bacterial cultures were adjusted to an OD_600_ of 0.2 in PBS and HeLa cells were infected with a multiplicity of infection (MOI) of 10. Assays were centrifuged for 5 min. at 500×g in order to synchronize infection and subsequently incubated for 25 min. at 37°C and 5% CO_2_ to allow host cell invasion by *Salmonella*. After infection, cells were washed trice with PBS and incubated with DMEM containing 10% FCS and 100 µg×ml^−1^ gentamicin for 1 h. The medium was replaced by DMEM containing 10% FCS and 10 µg×ml^−1^ gentamicin for the rest on the experiment.

### Transfection of HeLa cells and live cell imaging

About 2×10^4^ HeLa cells were seeded in 8 chamber slides (Nunc). The next day, HeLa cells were transfected with 500 ng plasmid DNA (pLAMP1-GFP) with the calcium-phosphate method [30]. Plasmid DNA was diluted with 250 mM CaPO_4_ this solution was mixed with a solution containing 1.4 mM phosphate, 140 mM NaCl, 50 mM Hepes, pH 7.05 and incubated for 1 min. at RT. This transfection mixture was added to cells in DMEM containing 10% FCS and incubated for 4 h. The medium was removed and cells were incubated with 10% glycerol for 1 min. at RT. Subsequently, the cells were incubated with DMEM with 10% FCS. Infection of the transfected cells was performed 16 to 20 h after transfection.

HeLa cells transfected with LAMP1-GFP were infected with various *Salmonella* strains at an MOI of 100. The infection was performed as described above. 1 h after infection, medium was replaced by DMEM-F12 with 10 µg×ml^−1^ gentamicin and 5 h after infection, live cell imaging was performed with a Zeiss Axiovert 200 M microscope with a Plan Apochromat 63 x/1.40 Oil Ph3 objective and an incubation chamber basically as described before [8].

### Immuno-fluorescence and image analyses

For immuno-staining, cells were grown on glass cover slips. 16 h post infection the cells were fixed with 3% para-formaldehyde (PFA) in PBS at RT for 15 min. For MTOC staining, cells were fixed in MeOH at −20°C. The antibodies were diluted in blocking solution containing 2% goat serum, 2% bovine serum albumin (BSA) and 0.1% saponin in PBS. The infected cells were stained with the various antibodies for 1 h at RT. Between the incubation steps the cells were washed thrice with PBS. The cover slips were mounted on Fluoprep (BioMérieux) and sealed with Entellan (Merck). Samples were analyzed using a Zeiss Axiovert 200 M wide-field microscope with an Axiocam MRm camera or a Leica SP5 confocal laser-scanning microscope (CLSM).

Infected and PFA-fixed cells were scored blindly for appearance of continuous LAMP2-positive tubular membrane compartments (SIF) or discontinuous, ‘beads-on-string’ like distribution of LAMP2-postive membranes (pseudo-SIF). Infected cells were either scored as positive for SIF or pseudo-SIF. Scoring of at least 100 infected cells per strain was performed in biological triplicates and quantitative interpretation of images a confirmed by a second investigator.

For quantification of SseF translocation, infected cells were immuno-stained for the HA-tag and *Salmonella* LPS. Selected cells are outlined and arbitrary units of pixel intensities for the fluorescence channels were determined using Axiovision 4.8 software.

Infected MeOH-fixed cells were scored for formation of microcolonies and distances of SCV to MTOC. Microcolonies were defined as cluster of at least 5 intracellular bacteria. The distance of intracellular bacteria to MTOC was performed on maximum projections and the linear distance to the proximal MTOC was measured using ImageJ (NIH).

### Selective permeabilization by digitonin treatment

In order to analyze the localization of the HA-epitope tagged C-terminus of WT SseF and various mutant variants, the digitonin permeabilization method describes by [31] was modified. The whole procedure was carried out on ice using ice-cold solutions. The infected cells were washed twice with KHM buffer (110 mM KAc, 20 mM Hepes pH 7.2, 2 mM MgAC) and then incubated for 5 min. in 10 µg×ml^−1^ digitonin (Fluka) in KHM buffer. The detergent was removed and the cells were incubated for 20 min. with KHM without digitonin to allow permeabilization. After a further washing step, cells were fixed with 3% PFA and subsequently immuno-stained in blocking solution without saponin.

### Subcellular fractionation

The subcellular fractionation and membrane extraction was performed with modifications as described elsewhere [32], [33]. About 2×10^7^ HeLa cells were infected with *sseF* [*sseF*::HA] with a MOI of 100 as described above. Cells were harvested 16 h p.i. and pellets were resuspended in homogenization buffer (3 mM imidazole, 250 mM sucrose, 0.5 mM EDTA, pH 7.4) containing complete protease inhibitor mix (Sigma). Cells were disrupted mechanically by vigorously passing the cells through a 22 G needle using a 1 ml syringe. Low speed centrifugation at 2,000×g for 20 min. was performed to pellet bacteria, unbroken cells, host nuclei and the cytoskeleton. This supernatant was centrifuged for 20 min. at 41,000×g to separate the cytoplasmic fraction (supernatant) from the membrane fraction (pellet).

To determine if SseF is associated with or integral in host cell membranes, the pellets were washed in membrane buffer (10 mM Tris-HCl, 5 mM MgCl_2_, pH 7.5) and then resuspended in high salt buffer (1 M KCl, 5 mM MgCl_2_), Triton X-100 buffer (0.1% Triton X-100, 5 mM MgCl_2_), or alkaline buffer (0.1 M Na_2_CO_3_, 5 mM MgCl_2_) and incubated on ice for 30 min. Insoluble substances were pelleted by centrifugation at 100,000×g for 30 min. Pellets were again washed in membrane buffer. The supernatants (extracted proteins) of the subcellular fractionation were precipitated over night at 4°C by addition of TCA to a final concentration of 15%. The samples were centrifuged at 10,000×g for 15 min. and pellets were washed in PBS. For immuno-blotting, the pellets were resuspended in 1 M Tris-HCl, pH 7.4 and diluted with 2× sample buffer.

## Supporting Information

Table S1 **Plasmids used in this study.**
(DOCX)

Table S2 **Oligonucleotides used in this study.**
(DOCX)
