# Supplementary material for: Functional Dissection of SseF, a Membrane-Integral Effector Protein of Intracellular Salmonella enterica
Source: PLoS One. 2012 Apr 18;7(4):e35004. doi: 10.1371/journal.pone.0035004 (PMC3329539; doi:10.1371/journal.pone.0035004)
Supplement: Table S1 — Plasmids used in this study. (DOCX) [file pone.0035004.s001.docx]

Table S2. Plasmids used in this study

| plasmid | relevant properties | reference |
| --- | --- | --- |
| pWSK29 | general cloning vector, low copy | [[1](#_ENREF_1)] |
| pBlueScript II SK(+) | general cloning vector, high copy | Stratagene |
| pKD46 | Temperature-sensitive replication, arabinose-inducible λ Red recombinase expression | [[2](#_ENREF_2)] |
| pCP20 | FLP expressing plasmid, temperature sensitive replication | [[2](#_ENREF_2)] |
| p*lamp-gfp* | *P*_cmv_ lamp1::gfp | lab collection |
| pFPV-mCherry | *P*_rpsM_::*cherry*, constitutively expressing *cherry* | [[3](#_ENREF_3)] |
| pFPV25.1 | *P*_rpsM_::gfpmut3, constitutively expressing gfp | [[4](#_ENREF_4)] |
| p2643 | pWSK29 *P*_s_*_seA_ sscB sseF*::HA | [[5](#_ENREF_5)] |
| p2644 | pWSK29 *P*_s_*_seA_ sscB sseF*sseG::HA | [[5](#_ENREF_5)] |
| p3122 | pWSK29 *P_sseA_ sscBsseF*::*sseG*::HA | lab stock |
| p3123 | pWSK29 *P_sseA_ sscBsseF*::*sseG*::HA | lab stock |
| p3248 | pWSK29 *P_sseA_ sscB sseF*_Δ179-189_::HA | this study |
| p3249 | pWSK29 *P_sseA_ sscB sseF*_Δ190-200_::HA | this study |
| p3250 | pWSK29 *P_sseA_ sscB sseF*_Δ201-212_::HA | this study |
| p3347 | pWSK29 *P_sseA_ sscB sseF*_Δ195-200_::HA | this study |
| p3348 | pWSK29 *P_sseA_ sscB sseF*_Δ195-205_::HA | this study |
| p3349 | pWSK29 *P_sseA_ sscB sseF*_Δ200-205_::HA | this study |
| p3351 | pWSK29 *P_sseA_ sscB* | this study |
| p3352 | pWSK29 *P_sseA_ sscB sseG*::*sseF*::HA | this study |
| p3356 | pWSK29 *P_sseA_ sscB sseG*::*sseF*_Δ240-277_::HA | this study |
| p3357 | pWSK29 *P_sseA_ sscB sseG*::*sseF*_Δ267-277_::HA | this study |
| p3358 | pWSK29 *P_sseA_ sscB sseG*::*sseF*_Δ291-338_::HA | this study |
| p3359 | pWSK29 *P_sseA_ sscB sseG*::*sseF*_Δ284-488_::HA | this study |
| p3360 | pWSK29 *P_sseA_ sscB sseG*::*sseF*_Δ346-352_::HA | this study |
| p3402 | pSK *P_sseA_ sscB sseF*::HA | this study |
| p3439 | pSK *P_sseA_ sscB sseF*^I201A^::HA | this study |
| p3440 | pSK *P_sseA_ sscB sseF*^I201A V204A^::HA | this study |
| p3441 | pSK *P_sseA_ sscB sseF*^I201A V204A L205A^::HA | this study |
| p3442 | pSK *P_sseA_ sscB sseF*_Δ206-212_::HA | this study |
| p3443 | pWSK29 *P_sseA_ sscBsseF*^I201A^::HA | this study |
| p3444 | pWSK29 *P_sseA_ sscBsseF*^I201A V204A^::HA | this study |
| p3445 | pWSK29 *P_sseA_ sscBsseF*^I201A V204A L205A^::HA | this study |
| p3446 | pWSK29 *P_sseA_ sscBsseF*_Δ206-212_::HA | this study |
| p3460 | pSK *gfp*-mut3 | this study |
| p3483 | pWSK29 *P_sseA_ sscB* N*sseG*::*sseF*::HA | this study |
| p3505 | pWSK29 *P_sseA_ sscB sseG*::*sseF*_Δ196-400_::HA | this study |
| p3506 | pWSK29 *P_sseA_ sscB NsseG*::*sseF*_Δ335-345_::HA | this study |
| p3508 | pWSK29 *P_sseA_ sscB NsseG::sseF*_Δ203-250_::HA | this study |
| p3509 | pWSK29 *P_sseA_ sscB NsseG::sseF*_Δ227-338_::HA | this study |
| p3538 | pSK *P_sseA_ sscB sseF*^I201R V204R L205R^::HA | this study |
| p3539 | pSK *P_sseA_ sscB sseF*^I201C V204C L205C^::HA | this study |
| p3540 | pWSK29 *P_sseA_ sscB* N*sseG*::*sseF*_Δ179-189_::HA | this study |
| p3541 | pWSK29 *P_sseA_ sscB* N*sseG*::*sseF*_Δ152-189_::HA | this study |
| p3542 | pWSK29 *P_sseA_ sscB* N*sseG*::*sseF*_Δ346-352_::HA | this study |
| p3543 | pWSK29 *P_sseA_ sscB sseF*^I201R-V204R-L205R^::HA | this study |
| p3544 | pWSK29 *P_sseA_ sscB sseF*^I201C-V204C-L205C^::HA | this study |

**References**

1. Wang RF, Kushner SR (1991) Construction of versatile low-copy-number vectors for cloning, sequencing and gene expression in *Escherichia coli*. Gene 100: 195-199.

2. Datsenko KA, Wanner BL (2000) One-step inactivation of chromosomal genes in *Escherichia coli* K-12 using PCR products. Proc Natl Acad Sci U S A 97: 6640-6645.

3. Drecktrah D, Knodler LA, Howe D, Steele-Mortimer O (2007) *Salmonella* trafficking is defined by continuous dynamic interactions with the endolysosomal system. Traffic 8: 212-225.

4. Valdivia RH, Hromockyj AE, Monack D, Ramakrishnan L, Falkow S (1996) Applications for green fluorescent protein (GFP) in the study of host-pathogen interactions. Gene 173: 47-52.

5. Kuhle V, Jäckel D, Hensel M (2004) Effector proteins encoded by *Salmonella* pathogenicity island 2 interfere with the microtubule cytoskeleton after translocation into host cells. Traffic 5: 356-370.
