# Supplementary material for: Functional Dissection of SseF, a Membrane-Integral Effector Protein of Intracellular Salmonella enterica
Source: PLoS One. 2012 Apr 18;7(4):e35004. doi: 10.1371/journal.pone.0035004 (PMC3329539; doi:10.1371/journal.pone.0035004)
Supplement: Table S2 — Oligonucleotides used in this study. (DOCX) [file pone.0035004.s002.docx]

#### Table S3. Oligonucleotides used in this study

| Name | Sequence (5’ – 3’) |
| --- | --- |
| SseF-Del-179-189-For | cctacagtttccactgcccgggagtgtaattacctccg |
| SseF-Del-179-189-Rev | cggaggtaattacactcccgggcagtggaaactgtagg |
| SseF-Del-190-200-For | gcggcctctttggacatgataggtgcggtactggattattg |
| SseF-Del-190-200-Rev | caataatccagtaccgcacctatcatgtccaaagaggccgc |
| SseF-Del201-212-For | cctccgttagcctgacggcgtctggcgacgatcaggaaaattctgttg |
| SseF-Del-201-212-Rev | caacagaattttcctgatcgtcgccagacgccgtcaggctaacggagg |
| SseF-Del-195-200-For | ggacatggggagtgtaattaccataggtgcggtactgga |
| SseF-Del-195-200-Rev | tccagtaccgcacctatggtaattacactccccatgtcc |
| SseF-Del-195-205-For | ggacatggggagtgtaattaccgattattgccttgcccgcccctc |
| SseF-Del-195-205-Rev | gaggggcgggcaaggcaataatcggtaattacactccccatgtcc |
| SseF-Del-200-205-For | gtaattacctccgttagcctgacggattattgccttgcccgcccctc |
| SseF-Del-200-205-Rev | gaggggcgggcaaggcaataatccgtcaggctaacggaggtaattac |
| SseF-Del-206-212-For | cggcgataggtgcggtactgtctggcgacgatcaggaaaattc |
| SseF-Del-206-212-Rev | gaattttcctgatcgtcgccagacagtaccgcacctatcgccg |
| SseG-EcoRI-For2 | cacgaattccgcatgaaacctgttagccca |
| SseGF-2(sseG-rev-stop) | ccgctgacggaatatgaattttctccggcgcacgttgttctg |
| SseGF-3(sseF-for-met) | cgccagaacaacgtgcgccggagaaaattcatattccgtcagcgg |
| HA-Rev-XbaI | ccatctagattaagcgtagtctggg |
| ProB-For-KpnI | ctaggtaccagaagagaacaacggcaag |
| SscB-rev-EcoRI-2 | cgcgaattcttaagcaataagagtatcaacc |
| SseGF-3(sseF-for-met) | cgccagaacaacgtgcgccggagaaaattcatattccgtcagcgg |
| SseGF-Fusion P2 | catttgggctaacaggtttcggttctccccgagatgtatga |
| SseGF-Fusion P3 | gaaacctgttagcccaaatg |
| SseF262-SseG1 SOE Rev | agcatttgggctaacaggtttcattggttctccccgagatgtatgatc |
